# Supplementary material for: The Clinical Impact of Metagenomic Next-Generation Sequencing (mNGS) Test in Hospitalized Patients with Suspected Sepsis: A Multicenter Prospective Study
Source: Diagnostics (Basel). 2023 Jan 16;13(2):323. doi: 10.3390/diagnostics13020323 (PMC9857658; doi:10.3390/diagnostics13020323)
Supplement: Supplementary file 1 [file diagnostics-13-00323-s001.zip › Supplemental file S1.pdf]

We listed all participating hospitals and cases of each hospital.

Participating hospitals:

The participating hospitals include Zhongshan Hospital of Fudan University (92 cases), Second Xiangya Hospital of Central South University (56 cases), The First Affiliated Hospital of Zhejiang University (15 cases), Tongji Hospital of Tongji Medical College, Huazhong University of Science and Technology (17 cases), The First Affiliated Hospital of Wenzhou Medical University (46 cases), The Second Affiliated Hospital of Suzhou University (27 cases), Qingdao Municipal Hospital (6 cases), Central Hospital of Minhang District, Shanghai (9 cases), and Shanghai Fifth People's Hospital (9 cases).
