# Supplementary material for: The Clinical Impact of Metagenomic Next-Generation Sequencing (mNGS) Test in Hospitalized Patients with Suspected Sepsis: A Multicenter Prospective Study
Source: Diagnostics (Basel). 2023 Jan 16;13(2):323. doi: 10.3390/diagnostics13020323 (PMC9857658; doi:10.3390/diagnostics13020323)
Supplement: Supplementary file 1 [file diagnostics-13-00323-s001.zip › Supplemental file S2.pdf]

The process of mNGS detection was explained in detail.

mNGS detection

Volume of 3-4 mL of blood were drawn from patients, placed in blood collection tube and stored at room temperature for 3-5 minutes before plasma separation and centrifuged at 4,000 rpm for 10 min at 4℃ within 8 h of collection. Plasma samples were transferred to new sterile tubes.DNA was extracted from 300 uL of plasma using the TIANamp Micro DNA Kit (DP316, TIANGEN BIOTECH ,Beijing, China) following the manufacturer's operational manual. The extracted DNA specimens were used for the construction of DNA libraries [1]. The total DNA were fragmented by Bioruptor® Pico (Diagenode, Belgium) to 150 bp. After fragmentation, poly A tail was added to the fragmented sequence for end repair purpose. Sample-specific adapter ligation and unbiased PCR was conducted for sample discrimination and sequence amplification. DNA libraries were purification with magnetic beads purification strategy using MGIEasy DNA purification kit (MGI, China) after a PCR amplification. The quality of the resulting library was measured by Qubit Fluorometer 3.0 (Invitrogen, USA) and Agilent 2100 Bioanalyzer (Agilent Technologies, USA). Qualified libraries (>2ng/uL, medium sequence size between 200-300 bp) were sequenced on BGISEQ-50 system [2]. At least 20 million reads were produced from each sample.

After removing low-quality, and short (length < 35 bp) reads, and computational

subtraction of human host sequences mapped to the human reference genome (hg19) using Burrows-Wheeler Alignment tool (version 0.7.10-r789)[3]. After removal of low-complexity reads using prinseq tool (version 0.20.3), the remaining data were classified by simultaneously aligning to the locally built reference databases, Microbial Genome Databases. The reference databases for microbial classification downloaded from NCBI contain 4,061 whole genome sequences of viral taxa, 2,473 bacterial genomes or scaffolds, 199 fungi and 135 parasites that are associated with human diseases. Data Analytics algorithms were used to exclude the microorganisms that were not significantly related to infection. Genus/species-specific reads uniquely aligned to infection-related microorganisms were reported.

The plasma samples were used to detect suspected pathogens causing sepsis, based on the theory that the nucleic acid of the pathogen will be released into the patient's plasma after infection. Therefore, the free nucleic acid in plasma could be used to detect bacteria/fungi. 300 ul of plasma ensured that enough DNA could be extracted and guaranteed the success of the test. In our test, no enrichment was used to enrich the DNA of bacterial/fungi. The sensitivity of bacterial/fungal detection was ensured by large volume of sequencing data (20M). Besides, the number of strictly aligned sequences of target microorganism detected at the genus/species level were recorded as reads

References:

1. Jeon YJ, Zhou Y, Li Y, Guo Q, Chen J, Quan S, et al. The feasibility study of non-

invasive fetal trisomy 18 and 21 detection with semiconductor sequencing platform. PLoS One. 2014;9:e110240. doi: 10.1371/journal.pone.0110240.

2. Li H, Durbin R. Fast and accurate short read alignment with Burrows-Wheeler transform. BIOINFORMATICS. 2009;25: 1754-60. doi: 10.1093/bioinformatics/btp324.
