# Supplementary material for: The Clinical Impact of Metagenomic Next-Generation Sequencing (mNGS) Test in Hospitalized Patients with Suspected Sepsis: A Multicenter Prospective Study
Source: Diagnostics (Basel). 2023 Jan 16;13(2):323. doi: 10.3390/diagnostics13020323 (PMC9857658; doi:10.3390/diagnostics13020323)
Supplement: Supplementary file 1 [file diagnostics-13-00323-s001.zip › Supplemental file S3.pdf]

Supplemental Table S1. The diagnosis efficacy comparison between mNGS and blood culture in different subgroups.

Subgroups	mNGS test		Blood culture	
	**Sensitivity**	**Specificity**	**Sensitivity**	**Specificity**
Age				
＞ 65 yrs	90.74%*	90.20%	37.78%	95.00%
⩽ 65 yrs	90.43%**	98.68%	35.00%	94.44%
Gender				
Male	90.48%**	94.32%	36.05%	95.45%
Female	90.70%**	97.44%	35.90%	92.31%
Shock index				
＞ 1.0	89.09%**	100.00%	34.00%	96.30%
⩽ 1.0	91.40%**	93.18%	37.33%	93.85%
Pulmonary infection				
Yes	92.0%**	92.68%	32.50%	92.59%
No	87.50%**	100%	42.22%	97.36%
Bloodstream infection				
Yes	96.15%*	92.86%	56.00%	90.91%
No	89.34%**	95.58%	31.00%	95.06%
Surgery/trauma history [a]				
Yes	95.83%**	97.78%	34.88%	97.22%
No	88.88%**	93.90%	36.59%	92.86%

Time from infection onset to enrollment				
≤ 5 days	91.23%**	96.30%	26.92%	95.45%
＞ 5 days	89.77%**	97.18%	41.67%	93.62%
Invasive operations before infection onset [b]				
Yes	89.61%**	98.53%	42.19%	91.53%
No	91.54%**	91.53%	29.51%	97.67%
Fever				
Yes	88.68%**	95.65%	40.45%	95.24%
No	95.24%**	94.29%	25.00%	93.10%
Altered mental status				
Yes	86.67%**	95.00%	41.54%	91.89%
No	94.37%**	95.52%	29.31%	96.36%
Antibiotics use before enrollment [c]				
Yes	90.70%**	94.96%	34.58%	94.05%
No	89.47%*	100%	44.44%	100%
ICU administration				
Yes	90.43%**	97.89%	35.58%	94.37%
No	90.91%*	87.50%	38.10%	95.24%
Sepsis				

Yes	89.13%**	98.57%	34.15%	93.88%
No	92.86%**	91.23%	39.53%	95.35%

a: surgery/trauma history within 3 months before enrollment; b: invasive procedures include punctures and drainages, tracheal intubation, urinary catheterization, arteriovenous catheterization, and superficial vein indwelling needle puncturing; c: antibiotics application within 2 weeks before enrollment.

Abbreviations: mNGS, metagenomic next-generation sequencing; ICU, Intensive care unit.

*P < 0.05, mNGS vs blood culture; **P < 0.001, mNGS vs blood culture.
