# Supplementary material for: The Clinical Impact of Metagenomic Next-Generation Sequencing (mNGS) Test in Hospitalized Patients with Suspected Sepsis: A Multicenter Prospective Study
Source: Diagnostics (Basel). 2023 Jan 16;13(2):323. doi: 10.3390/diagnostics13020323 (PMC9857658; doi:10.3390/diagnostics13020323)
Supplement: Supplementary file 1 [file diagnostics-13-00323-s001.zip › Supplemental file S4.pdf]

Supplemental Table S2. Univariate linear regression of inflammatory factors level and reads of responsible pathogens in mNGS reports.

Inflammatory Factor	P-value	B (95% CI)	R-Square
IL-1β	0.024	0.349 (0.051, 0.647)	0.182
Eotaxin	0.004	0.465 (0.157, 0.773)	0.247
IFN- α	< 0.001	2.629 (1.450, 3.809)	0.436
HGF	0.761	0.039 (-0.221, 0.299)	0.003
TNF- α	< 0.001	0.905 (0.503, 1.307)	0.432
IL-9	0.149	0.415 (-0.158, 0.988)	0.070

Abbreviations: mNGS, metagenomic next-generation sequencing; B, standardized linear regression coefficient; IL, interleukin; IFN, interferon; HGF, hepatocyte growth factor; TNF, tumor necrosis factor.
