# Supplementary material for: The Clinical Impact of Metagenomic Next-Generation Sequencing (mNGS) Test in Hospitalized Patients with Suspected Sepsis: A Multicenter Prospective Study
Source: Diagnostics (Basel). 2023 Jan 16;13(2):323. doi: 10.3390/diagnostics13020323 (PMC9857658; doi:10.3390/diagnostics13020323)
Supplement: Supplementary file 1 [file diagnostics-13-00323-s001.zip › Supplemental file S5.pdf]

Supplemental Table S3. Univariate linear regression of inflammatory factors level and reads of pathogens in sepsis group.

Inflammatory factor	P-value	B (95% CI)	R-square
IL-1β	0.070	0.342(-0.031, 0.714)	0.171
Eotaxin	0.019	0.519(0.097, 0.941)	0.259*
IFN- α	0.002	2.822(1.209, 4.435)	0.429*
HGF	0.984	0.003(-0.343, 0.349)	0.000
TNF- α	0.002	0.958(0.419, 1.496)	0.437*
IL-9	0.077	0.782(-0.095, 1.658)	0.155

Abbreviations: B, standardized linear regression coefficient; IL, interleukin; IFN, interferon; HGF, hepatocyte growth factor; TNF, tumor necrosis factor. * represents the significance of R value < 0.05.
